# Prefrontal dopamine and behavioral flexibility: shifting from an “inverted-U” toward a family of functions

**DOI:** 10.3389/fnins.2013.00062

**Published:** 2013-04-19

**Authors:** Stan B. Floresco

**Affiliations:** Department of Psychology, Brain Research Centre, University of British ColumbiaVancouver, BC, Canada

**Keywords:** prefrontal, dopamine, D_1_, D_2_, set-shifting, decision making, microdialysis, rats

## Abstract

Studies on prefrontal cortex (PFC) dopamine (DA) function have revealed its essential role in mediating a variety of cognitive and executive functions. A general principle that has emerged (primarily from studies on working memory) is that PFC DA, acting on D_1_ receptors, regulates cognition in accordance to an “inverted-U” shaped function, so that too little or too much activity has detrimental effects on performance. However, contemporary studies have indicated that the receptor mechanisms through which mesocortical DA regulates different aspects of behavioral flexibility can vary considerably across different DA receptors and cognitive operations. This article will review psychopharmacological and neurochemical data comparing and contrasting the cognitive effects of antagonism and stimulation of different DA receptors in the medial PFC. Thus, set-shifting is dependent on a co-operative interaction between PFC D_1_ and D_2_ receptors, yet, supranormal stimulation of these receptors does not appear to have detrimental effects on this function. On the other hand, modification of cost/benefit decision biases in situations involving reward uncertainty is regulated in complex and sometimes opposing ways by PFC D_1_ vs. D_2_ receptors. When viewed collectively, these findings suggest that the “inverted-U” shaped dose-response curve underlying D_1_ receptor modulation of working memory is not a one-size-fits-all function. Rather, it appears that mesocortical DA exerts its effects via a family of functions, wherein reduced or excessive DA activity can have a variety of effects across different cognitive domains.

Brozoski et al. (1979) originally reported that depletion of dopamine (DA) in the prefrontal cortex (PFC) of monkeys impaired delayed responding in a manner comparable to complete removal of the frontal lobes. These seminal findings have since sparked a substantial amount of psychopharmacological, neurophysiological, and computational research on how mesocortical DA regulates complex forms of cognition. Much of the work stemming from these initial findings has focused on processes related to working memory, revealing that these functions are dependent primarily on PFC D_1_ receptor activity. A particularly influential discovery from this line of research is that PFC D_1_ receptor modulation of working memory takes the form of an “inverted-U” shaped curve (Arnsten, 1997; Zahrt et al., 1997; Williams and Castner, 2006), where suboptimal or excessive D_1_ activity can have detrimental effects on cognition.

The notion that normal PFC functioning is dependent on an optimum range of DA activity, whereas “too little” or “too much” D_1_ receptor stimulation has detrimental effects on working memory has become a cornerstone of our understanding of how mesocortical DA regulates cognition. However, the frontal lobes regulate a variety of other functions distinct from working memory, such as cognitive flexibility, cost/benefit decision making, and emotional processes. More contemporary studies have begun to elucidate how PFC DA may regulate these functions, and an emerging impression is that PFC DA regulation of these other functions differs considerably from mechanisms that facilitate working memory.

DA exerts its effects on PFC neural activity via multiple receptor subtypes. Both D_1_-like and D_2_-like (D_2_, D_4_) receptors are expressed within the PFC, although the subcellular localization of these receptors differs. Expression of D_1_ receptors on principle pyramidal neurons appears to be substantially greater than D_2_ receptors (Gaspar et al., 1995), whereas both types of receptors have been localized on GABAergic interneurons and may also reside on presynaptic excitatory glutamate terminals (Sesack et al., 1995; Mrzijak et al., 1996; Muly et al., 1998; Wedzony et al., 2001). Numerous studies have shown that activation of D_1_, D_2_, or D_4_ receptors exerts complex and dissociable electrophysiological actions on the activity of different classes of PFC neurons that may either increase or decrease the excitability of these cells and differentially modulate PFC neural network activity, depending of a variety of factors (see Seamans and Yang, 2004 for a review). Moreover, recent studies have indicated that there may be separate population of PFC pyramidal neurons that preferentially express only D_1_ or D_2_ receptors (Gee et al., 2012; Seong and Carter, 2012). These anatomical and neurophysiological findings suggest that DA may exert differential effects on the activity of PFC neural networks which in turn may subserve a variety of distinct cognitive operations. Yet, despite these findings, the majority of studies on the role of PFC DA in functions such as working memory have focused on the role of D_1_ receptors, whereas until recently, the functional role of D_2_ and D_4_ receptors has been less clear. This review will highlight some recent advances in our understanding of how PFC DA regulates a variety of executive functions, focusing primarily on psychopharmacological and neurochemical data obtained from rodents, with an emphasis on the differences in the principles of operation through which medial PFC DA regulates different cognitive domains.

## DA, the “inverted-U” and working memory: important caveats

One of the earliest and direct demonstrations that supranormal stimulation of PFC D_1_ receptors can perturb working memory came from the seminal study by Zahrt et al. (1997). They showed that infusions of the full D_1_ agonist SKF 81297 (0.01–0.1 μg) in the prelimbic region of the medial PFC of rats dose-dependently impaired delayed alternation on a T-maze task. An influential aspect of this paper was a summary figure, showing that treatment with a D_1_ agonist or antagonist (SCH 23390) markedly reduced the proportion of correct responses when compared to control conditions or combined agonist/antagonist treatment. What was particularly striking about this synthesis was how actual empirical data were plotted to clearly demonstrate an “inverted-U” shaped function underlying dopaminergic modulation of working memory. However, an important point that is often overlooked is that impairments in delayed alternation induced by D_1_ antagonism (the “too little” end of the curve) have been observed after *systemic* D1 receptor blockade. In contrast, a subsequent study using a near-identical task found that blockade of either D_1_ or D_2_ receptors in the medial PFC *did not impair* delayed alternation (Romanides et al., 1999). This discrepancy between the effects of systemic vs. local manipulations of DA activity indicates that caution is warranted when attributing the specific neural loci where systemic drug treatments may be acting to affect behavior and cognition. Note that in the aforementioned study, blockade of glutamate receptors did impair performance, indicating that working memory assessed in this manner is dependent on the integrity of excitatory transmission in the PFC. Yet, the fact that blockade of DA receptors in the rat medial PFC did not impair delayed alternation suggests that this form of delayed responding is not a particularly sensitive paradigm for assessing PFC DA regulation of working memory functions in rodents. Moreover, it suggests that certain aspects of working memory dependent on the PFC may nevertheless be relatively insensitive to reductions in mesocortical DA. This is in keeping with studies in primates showing that performance of a self-ordered sequencing task or a spatial delayed response task were both impaired by excitotoxic lesions of the PFC, yet PFC DA depletion only impaired delayed responding and left self-ordered working memory intact (Collins et al., 1998).

**Figure 1:**
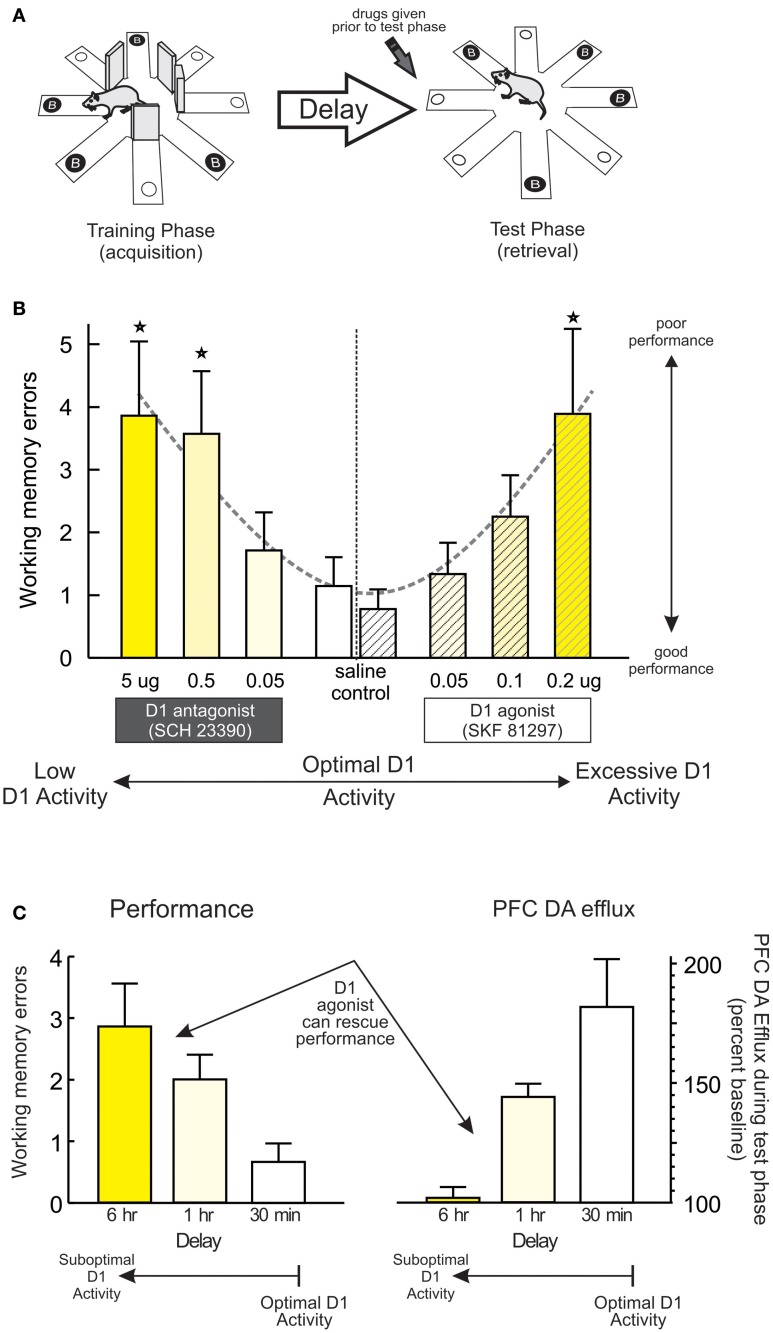
**PFC D1 receptor modulation of working memory. (A)** A delayed response variant of the radial-arm maze task used to collect data presented in subsequent panels. The task consists of a training (acquisition) and a test (retrieval) phase. During the training phase, the rat must retrieve food from four randomly selected arms, with the other arms blocked. During a test phase occurring after a delay, arms that were blocked previously are now open and baited. DA drugs were administered prior to the test phase. **(B)** Infusions of a D_1_ antagonist dose-dependently impaired working memory performance on this delayed-response task. Similarly, treatment with the D1 agonist SKF 81297 also impaired performance when infusions were made after a relatively short delay (30 min). These data have be re-plotted from those originally reported by Seamans et al. (1998) and Floresco and Phillips (2001) to highlight the effects of reduced vs. excessive D1 activation on performance. For this and all subsequent figures, dashed lines emphasize the dose-response function associated with reduced or excessive DA receptor stimulation. In the case of working memory, the effects present as a classic the U-shaped function, where reduced or excessive PFC D1 activity caused poorer performance relative to control conditions, numbers underneath each bar represents drug dose (in μg), and stars represent *p* < 0.05 vs. relative control treatments or groups. **(C)** Behavioral performance and peak increase in PFC DA efflux observed during the test phase of this task following a typical 30 min, or extended 1 or 6 h delays. Left panel shows that extending the delay period degrades performance and results in more working memory errors. Right panel shows that in these same animals, poorer performance was associated with reduced PFC DA efflux. Under these conditions, infusions of a D1 agonist can rescue performance. Adapted from Phillips et al. (2004).

Another important principle underlying PFC DA modulation of working memory is the relative baseline levels of performance. Work by our group has used a delayed response variant of the radial-arm maze task (Figure 1A) utilizing a comparatively long delay (30 min) that, unlike delayed alternation, is sensitive to blockade of PFC D_1_ (but not D_2_) receptors (Seamans et al., 1998; Figure 1B, left; Figure 5A). We exploited this procedure to manipulate baseline performance by testing separate group of rats after either a typical, 30 min delay (when performance was good) or after an extended, 12-h delay (which degrades performance in control animals) (Floresco and Phillips, 2001). In keeping with previous findings, intra-PFC (prelimbic) infusion of the D_1_ agonist SKF 81297 (0.05–0.2 μg) dose-dependently impaired working memory after the 30 min delay, compared to control rats that showed near-optimal performance (Figure 1B, right; Figure 5A). In contrast, control rats subjected to an extended 12 h delay made considerably more errors, presumably because the memory for the expected location of reward had degraded during this period. What was striking was that, under these conditions where performance was degraded, treatment with the same doses of the D_1_ agonist had the diametrically opposite effect to that observed when performance was good, in that these treatments improved performance relative to controls. Similar results have been obtained with the same agonist using a within-subjects design in combination with a delayed-response task incorporating shorter delays (Chudasama and Robbins, 2004). Thus, pharmacological stimulation of PFC D_1_ receptors does not always impair working memory, and can actually improve performance following degradation of the memory that the subject must “work” with (e.g., after longer delays). Note that degradations in performance induced by longer delays have been associated with reduced levels of mesocortical DA efflux compared to conditions where performance is good (Phillips et al., 2004; Figure 1C). Thus, differential effects of PFC D_1_ stimulation on working memory may be mediated in part by the relative levels of mesocortical DA transmission, with good vs. poor performance linked to higher vs. lower levels of DA efflux. Under these conditions, exogenous stimulation of PFC D_1_ receptors would either be expected to overstimulate these receptors (and impair good performance) or normalize levels of D_1_ activity and improve performance, in keeping with the idea of the inverted-U shaped function.

Unlike PFC D_1_ receptors, blockade of D_2_ receptors has repeatedly been shown to not disrupt working memory in primates or rats (Sawaguchi and Goldman-Rakic, 1994; Seamans et al., 1998; Romanides et al., 1999), even though local application of D_2_ agonists or antagonists augments or attenuates “response”-related firing of PFC neurons in monkeys performing an occulomotor delayed response task (Wang et al., 2004). Although the effects of PFC D_2_ receptor stimulation on working memory performance have yet to be explored fully, one notable study revealed that prelimbic PFC infusions of a D_2_ agonist disrupts delayed responding on a U-maze, whereas PFC D_2_ antagonism reduced proactive interference (Druzin et al., 2000). Thus, under some conditions, PFC D_2_ receptor modulation of working memory may take the form of a monotonic function (i.e., lower/higher levels of D_2_ activation associated with better/poorer performance), in a manner that is distinct and antagonistic to the inverted-U shaped function underlying D_1_ receptor modulation. However, as discussed below, the principles of operation through which different DA receptors interact to regulate other executive processes mediated by the frontal lobes can differ considerably from those underlying working memory.

## Prefrontal DA and behavioral flexibility

Another key function of the mammalian PFC is to facilitate alterations in behavior in response to changing environmental demands (Dias et al., 1996; Brown and Bowman, 2002; Floresco et al., 2009). Behavioral flexibility is not a unitary phenomenon, but rather, may be viewed as a hierarchical process, ranging from simpler to more complex processes that are subserved by anatomically-distinct prefrontal and subcocortical regions. For example, extinction entails the suppression of a conditioned response elicited by a stimulus that no longer predicts reinforcement. Although the contribution of mesocortical DA to this form of flexibility remains to be characterized thoroughly, there have been reports that D_2_ and D_4_ receptors in the infralimbic medial PFC, may facilitate consolidation of fear extinction memories (Pfeiffer and Fendt, 2006; Mueller et al., 2010).

Reversal learning is a more complex form of flexibility engaged when an organism must discriminate between two or more stimuli, only one of which is associated with reinforcement. Reversal shifts require a switch between stimulus-reinforcement associations within a particular stimulus dimension (i.e., use the same basic strategy, but approach a different stimulus), a form of flexibility critically-dependent on the orbitofrontal PFC in both primates and rats (Dias et al., 1996, 1997; McAlonan and Brown, 2003). Unlike other forms of flexibility, reversal learning is generally unimpaired by global depletion of PFC DA (Roberts et al., 1994; Crofts et al., 2001). Rather, serotonin inputs to the orbital PFC appears to be the monoamine neurotransmitter that is of primary importance in modulating reversal learning (Clarke et al., 2004, 2005), although DA input to striatal regions also facilitates this form of flexibility (O'Neill and Brown, 2007; Clarke et al., 2011).

On the other hand, shifts between strategies, rules or attentional sets taps into higher-order cognitive functions, requiring attention be focused to multiple aspects of complex environmental stimuli. In humans, an inability to shift strategies is epitomized by impairments on the Wisconsin Card Sorting task. Patients with frontal lobe damage are initially able to sort cards by one dimension (e.g., color), but have great difficulty in altering their strategy when required to organize cards by another dimension, (number or shape), perseverating to the now incorrect strategy. Studies with laboratory animals have revealed that lesions/inactivation of the lateral PFC in primates or the medial PFC in rats do not affect initial discrimination learning, but profoundly impair the ability to inhibit an old strategy and utilize a new one (Dias et al., 1996, 1997; Ragozzino et al., 1999; Brown and Bowman, 2002; Floresco et al., 2008a), even though these manipulations do not affect reversal learning.

Much of the research on how mesocortical DA modulates behavioral flexibility has focused on attentional or strategy set-shifting. An initial report by Roberts et al. (1994) used an intradimensional/extradimensional (ID/ED) shifting task, wherein marmosets conducted a series of two-choice discriminations using complex stimuli (e.g., sets of lines overlaid onto different shapes). During the initial phases, subjects discriminated stimuli based on one stimulus dimension (e.g., lines), but during the critical ED phase, they had to shift their attention to the other stimulus dimension. Depletion of PFC DA actually improved ED set shifting, even though these manipulations disrupted working memory assessed with a spatial delayed-response task. The improvement in set shifting was later attributed to a disruption in attentional set formation, as a subsequent study showed that PFC DA depletion impaired repeated ID shifts within the same stimulus dimension (Crofts et al., 2001). However, this effect was only observed for one type of ED shift when animals were required to shift responding from a more difficult “lines” dimension to a “shapes” dimension. Nevertheless, these data indicate that mesocortical DA serves to stabilize representations, facilitating the ability to attend to relevant stimuli (Robbins, 2005; Robbins and Arnsten, 2009).

**Figure 2:**
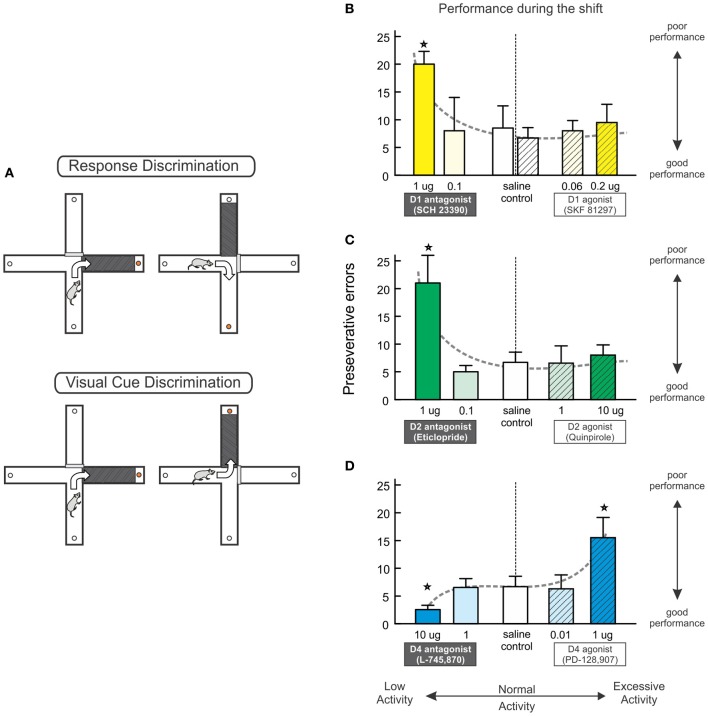
**Multiple PFC DA receptors regulate set shifting. (A)** The set shifting task conducted on a cross-maze requires rats to initially learn one discrimination rule (e.g., always turn right) to receive food reinforcement (top). A visual-cue insert is randomly placed in one of the arms but does not reliably predict reward. During the set-shift (bottom), the rat is now required to use a visual-cue discrimination strategy, necessitating a shift from the old strategy and approach toward the previously-irrelevant cue. **(B–D)** The effects of blockade and stimulation of D_1_, D_2_, and D4 receptors on set shifting. These data have be re-plotted from those originally reported by Ragozzino (2002) and Floresco et al. (2006) to highlight the effects of reduced vs. excessive DA receptor activation on perseverative errors made during the shift. Blockade of PFC D1 **(B)** or D2 **(C)** receptors significantly impairs strategy set-shifting, whereas pharmacological stimulation of these receptors did not affect performance. **(D)** Blockade of D_4_ receptors improves performance, whereas D_4_ stimulation impaired set shifting. Stars represent *p*<0.05.

One way to assesses set-shifting ability in rodents that is amenable to psychopharmacological investigation is with a strategy-shifting task conducted either on a cross-maze or in an operant chamber. Rats initially learn to use either an egocentric response (e.g., always turn left) or visual-cue discrimination strategy (e.g., always approach the arm with a visual cue, located in the left, or right arm with equal frequency) to obtain reinforcement (see Figure 2A). During the shift, rats must cease using the previously-acquired strategy and learn the alternative discrimination. As has been observed with studies using ID/ED shifting tasks designed for rodents, strategy set-shifting is disrupted by inactivation of the medial, but not orbital PFC (Ragozzino et al., 1999; Birrell and Brown, 2000; McAlonan and Brown, 2003; Floresco et al., 2008a; Ghods-Sharifi et al., 2008). Another advantage of the strategy shifting task is that it permits a detailed analysis of the different types of errors committed during the shift, providing insight into whether impairments are due to enhanced perseverative responding or a deficit in acquiring or maintaining new strategies. Reversible inactivation of the medial PFC causes robust perseverative-type deficits when rats must shift from one strategy to another (Ragozzino et al., 1999; Floresco et al., 2008a).

Psychopharmacological studies have revealed some similarities, but also important differences in the receptor mechanisms through which PFC DA regulates set-shifting viz à viz working memory. Thus, akin to its important for working memory, medial prelimbic PFC D_1_ receptor activity also facilitates strategy set-shifting, as blockade of these receptors with SCH 23390 induces severe perseverative deficits (Ragozzino, 2002, Figure 2B, left). However, a subsequent series of experiments by our group (Floresco et al., 2006) revealed blockade of PFC D_2_ receptors also enhanced perseveration during set-shifting, indicating that, unlike working memory, this form of behavioral flexibility is critically-dependent on a cooperative interaction of both D_1_ and D_2_ receptors in the PFC (Figure 2C, left).

Further, dissociations in the DA receptor pharmacology underlying working memory and set-shifting were observed following administration of DA agonists into the prelimbic medial PFC. Infusions of the D_1_ agonist, SKF 81297 (at doses known to affect working memory performance) neither impaired nor improved set-shifting (Figure 2B, right). Note that the lack of effect of PFC D_1_ stimulation is in keeping with findings from another study, where infusion of the agonist SKF 38393 did not alter set-shifting on an ID/ED task, although these manipulations did ameliorate impairments induced by repeated amphetamine (Fletcher et al., 2005). Similar to the lack of effect with a D_1_ agonist, intra-PFC application of the D_2_ agonist quinpirole also did not affect set-shifting (Figure 2C, right, and also see Figure 5B). Another interesting observation was that, unlike D_1_ and D_2_ receptors, PFC D_4_ receptor modulation of set-shifting took the form of a negative sigmoidal function, as stimulation of these receptors impaired performance, and their blockade improved shifting relative to controls (Figure 2D). Collectively, these findings indicate that the construct of an “inverted-U” shaped function underlying D_1_ (or D_2_/D_4_) receptor modulation of working memory does not appear to hold true for set-shifting functions mediated by the PFC. In this regard, it is plausible that combined stimulation of both of these receptors may have beneficial effects on set-shifting, as systemic treatment with the COMT inhibitor tolcapone has been reported to selectively increase PFC DA efflux and improve ED shifting (Tunbridge et al., 2004).

Additional insight into the contributions of PFC DA to set shifting comes from *in vivo* microdialysis studies conducted in freely-behaving rats performing a strategy set-shifting task similar to the one described above (Stefani and Moghaddam, 2006). These experiments also included two key control groups, the first being a yoked-reward group, where rats obtained reward on an intermittent schedule matched to rats performing the task but were not required to discriminate between arms or switch strategies. Thus, in this group, any choice led to either reward or no reward in accordance with a pattern of reinforcement experienced by a rat that actually performed the set-shifting task. However, in this instance, the specific response-reward contingencies were unpredictable from the perspective of the animals in this group. A second, reward-retrieval condition had rats obtain food on every trial, regardless of their choice.

The investigators observed that for rats trained on the set-shifting task, extracellular levels of PFC DA increased during learning of the initial rule, even though intact PFC DA does not appear to be necessary for learning simple discriminations (Ragozzino, 2002). When rats had to shift to a different rule in conflict with the first (a process that *is* dependent on PFC DA activity), PFC DA levels increased again, with a magnitude comparable to that observed during performance of a working memory task on a radial maze (~80–100% above baseline; Phillips et al., 2004). Importantly, for rats trained on the set-shifting task, the relationship between PFC DA levels and performance during the shift did not reflect an “inverted-U” type function (i.e., moderate increases in DA associated with better performance compared to higher or lower levels). Instead, the relationship between the magnitude of DA efflux and behavioral performance was curvilinear, in that higher levels PFC DA efflux was associated with better performance during the shift (i.e., fewer trials required to achieve criterion performance). This finding is in keeping with the observation that pharmacological increases in PFC DA activity do not impair set-shifting, and may actually facilitate these functions in some situations. Interestingly, rats in the yoked-reward group (but not reward-retrieval group) displayed a profile of DA release similar to that observed in rats actually performing the set shift, despite the fact that the scheduling of reinforcement in this condition did not permit them to learn any reliable response-reward contingencies. This latter finding suggests that PFC DA transmission is particularly sensitive to situations where reward availability is unpredictable or uncertain. This increase in PFC DA transmission triggered by unexpected reward deliveries or omissions may serve as a signal that reward contingencies are changing and promote adaptations in behavior. Indeed, as will be discussed below, recent findings have shown decision making involving reward uncertainty is modulated in a particularly complex way by different DA receptors in the PFC.

## Prefrontal DA and cost/benefit decision making

Since, the pioneering work of Damasio and colleagues showing that patients with damage to the ventromedial PFC were impaired on tasks designed to simulate real-life decisions in terms of uncertainty, reward and punishment (Bechara et al., 1994, 1999), there has been a growing interest in the neural circuitry underlying different forms of cost/benefit decision making. These types of situations require coordination of various cognitive and motivational processes to ensure that a decision maker adjusts choice biases in a flexible manner when cost/benefit contingencies change. A key component of decision making that can be assessed in rodents is the evaluation of costs associated with different actions relative to the rewards that may be obtained by those actions. In these studies, animals typically choose between smaller, readily-available rewards, or a larger/more palatable reward associated with some form of cost which can diminish the subjective value of objectively larger or more-preferred rewards. All things being equal, animals typically choose more (or “better”) vs. less food, yet, imposition of certain costs lead to a “discounting” of preferred rewards. Costs that are effective in biasing choice behavior include (1) delays to reward delivery, (2) requiring animals to exert greater physical effort to obtain the reward, or (3) making reward delivery probabilistic (i.e., uncertain/risky).

Over the last 10 years, studies in rats have shown that different forms of cost/benefit decision making are regulated by anatomically-distinct regions of the frontal lobes, with the lateral orbital PFC playing a greater role in delay-related judgments, the dorsal anterior cingulate region of the medial PFC contributing to effort based decision making, and the prelimbic region of the medial PFC facilitating risk/reward judgments when reward probabilities are volatile (Walton et al., 2003; Winstanley et al., 2004; Rudebeck et al., 2006; St. Onge and Floresco, 2010; Zeeb et al., 2010). Although, each of these forms of decision making are sensitive systemic manipulations of DA transmission (Floresco et al., 2008b), there has been relatively little work on how mesocortical DA transmission regulates these decisions. Blockade of D_1_ (but not D_2_) receptors in the anterior cingulate reduces preference for larger rewards associated with a greater effort cost (Schweimer and Hauber, 2006). On the other hand, blockade of D_1_ or D_2_ receptors in the orbital PFC, or administration of D_1_ receptor agonists or antagonists into the medial PFC increases delay discounting (Loos et al., 2010; Zeeb et al., 2010).

**Figure 3:**
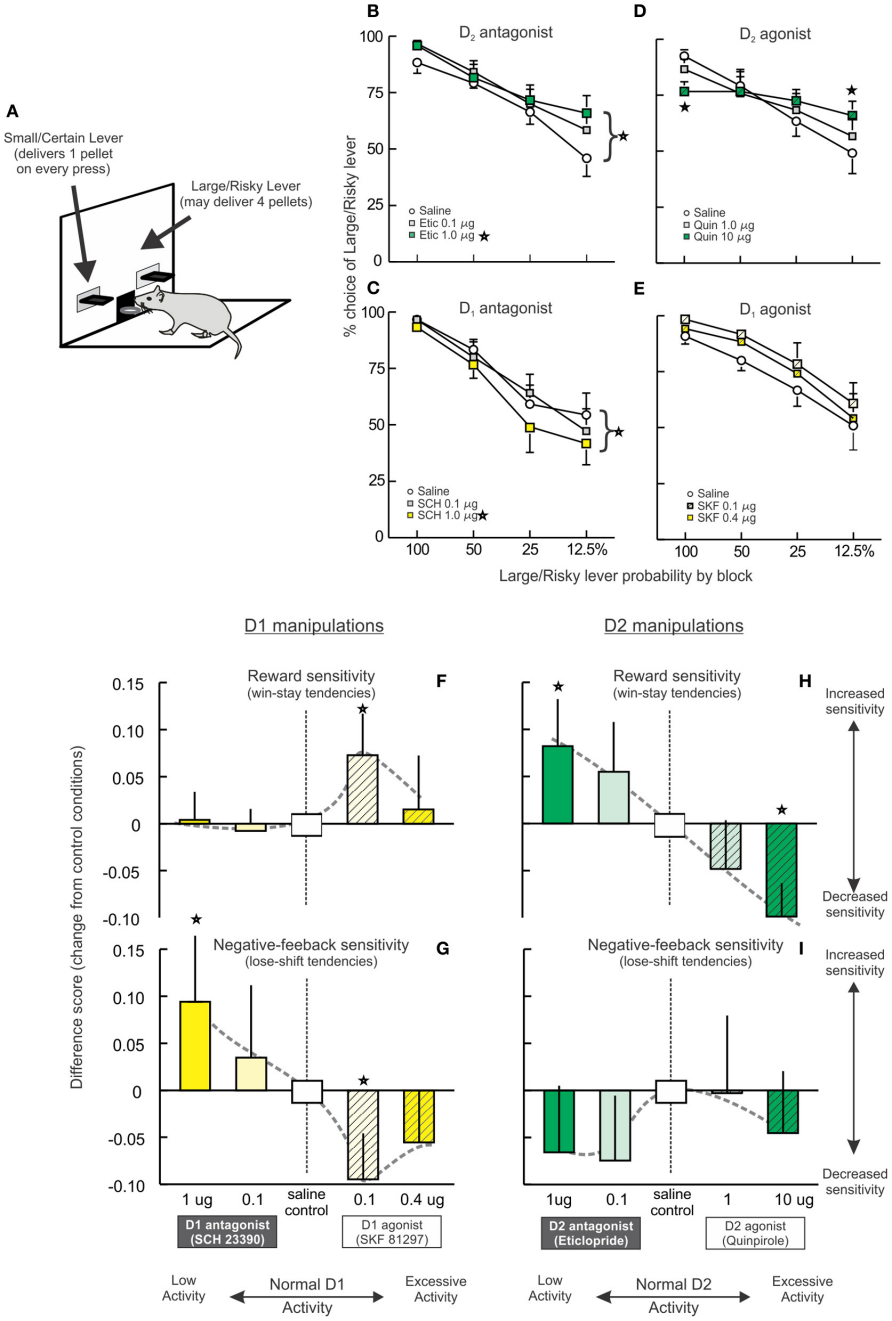
**Opposing effects of PFC D_1_ and D_2_ receptor manipulations on risk-based decision making. (A)** The probabilistic discounting task required rats to choose between a small/certain reward option or a large/risky option. The probability of obtaining the larger reward changes in a systematic manner over blocks of free-choice trials. **(B–E)** Effects of PFC DA receptor manipulations on probabilistic discounting. Data are plotted in terms of percentage choice of the Large/Risky lever during free choice trials by probability block. **(B)** Blockade of D2 receptors retarded discounting and increased risky choice. **(C)** In contrast, blockade of PFC D1 receptors accelerated probabilistic discounting, reducing risky choice. **(D)** The D_1_ agonist SKF 81297 induced a slight, non-significant increase in risky choice. **(E)** Infusions of the D_2_ agonist quinpirole abolished discounting, decreasing risky choice during the initial block and increasing choice during the final block. **(F–I)** Effects on reward and negative-feedback sensitivity, indexed by win-stay and lose-shift ratios. For clarity and comparative purposes, the data are presented as difference scores between the ratios obtained on drug vs. control treatments (positive values indicate an increased ratio, negative values a decrease after drug treatment relative to control treatments). Adapted from St. Onge et al. (2011). Stars represent *p*<0.05.

Work by our group has investigated the contribution of the rat prelimbic PFC to certain components risk-based decision making using a probabilistic discounting task, wherein rats choose between two options; a smaller, certain reward (1-pellet) or a larger uncertain (risky) option that may or may not yield 4-pellets (Figure 3A). The probability of obtaining the larger reward changes in a systematic manner over blocks of discrete, free-choice trials, ranging from 100 to 12.5%. Note that no explicit cues that signal changes in the odds of obtaining the larger reward are provided. Thus, in order to adjust their decision biases in an effective manner, rats must use internally-generated information to keep track of actions and outcomes (rewarded vs. non-rewarded choices) over multiple trials. This aspect of reward monitoring is dependent on the medial PFC, as inactivation of this region severely disrupts the ability to modify choice biases when reward probabilities change (St. Onge and Floresco, 2010). When the odds of obtaining the larger reward are initially good (100%) and gradually diminish over a session, PFC inactivation impairs shifting of decision biases toward the smaller/certain option in well-trained rats, which in this case results in an apparent increase in risky choice. Conversely, when the odds are initially poor (12.5%) and then increase, PFC inactivation retards shifts in bias toward the large/risky option, resulting in an overall decrease in risky choice. Thus, the medial PFC appears to play a critical role in detecting and tracking changes in action/outcome contingencies and reward availability, which in turn facilitates modifications in choice behavior when reward probabilities change.

We investigated the contribution of different DA receptors in the prelimbic medial PFC to this form of decision making, using doses of agonists and antagonists known to differentially affect working memory and set-shifting (St. Onge et al., 2011). Blockade of D_2_ receptors with eticlopride induced an effect similar to PFC inactivation, impairing shifts in choice biases as reward probabilities decreased over time, which in this experiment manifested as an increase in risky choice (Figure 3B). Thus, D_2_ receptor modulation of PFC neural activity facilitates modifications of decision biases in response to changes in risk/reward contingencies. In stark contrast, antagonism of PFC D_1_ receptors with SCH 23390 induced the opposite effect of D_2_ blockade (and PFC inactivation), causing a decrease in risky choice (Figure 3C). Thus, it appears that in some circumstances, D_1_ and D_2_ receptors regulate distinct and seemingly opposing functions related to risk-based decision making. Although, the mechanisms through which blockade of D_1_ vs. D_2_ receptors may induce opposing changes in behavior remains to be clarified, these effects may be related in part to actions of these receptors on separate populations of PFC pyramidal neurons (Gee et al., 2012; Seong and Carter, 2012), or their differential effects on the network activity of PFC neuronal populations (Durstewitz et al., 2000; Seamans and Yang, 2004).

Intra-PFC infusions of a D_1_ agonist altered decision making in a manner symmetrical to D_1_ blockade, inducing a moderate increase in risky choice that was not statistically-significant. Interestingly, these effects were numerically greater after treatment with the lower dose of SKF 81297 (0.1 μg) compared to the higher dose (0.4 μg; Figure 3E). A more pronounced disruption in decision making was induced by D_2_ receptor stimulation with quinpirole. These treatments markedly flattened the discounting curve, as rats displayed no discernible discounting upon changes in reward probabilities (Figure 3D). Thus, excessive D_2_ receptor activation severely interfered with the ability to adjust choice, causing rats to employ a simpler alternation strategy while maintaining a bias toward the large/risky option. This finding, in combination with the effects of eticlopride, suggests that the relative levels of both D_1_ and D_2_ receptor tone in the medial PFC has a critical impact on this aspect of decision making and either increasing or decreasing activity at either receptor interferes with performance.

Further difference in PFC D_1_/D_2_ modulation of different aspects of risk/reward decision making were unveiled upon examination of changes in reward and negative-feedback sensitivity induced by these treatments. Reward sensitivity was assessed by measuring the proportion of trials where subjects followed a risky “win” with another risky choice (a.ka., win-stay ratios), whereas, sensitivity to reward omissions was indexed by proportion of trials where rats shifted to the small/certain option after a non-rewarded risky choice (i.e., lose-shift ratios). Under control conditions, rats followed a risky win with another risky choice on 80–90% of these types of trials. Conversely, when rats played risky and were not rewarded, they chose the small/certain option on 25–30% of subsequent trials. Both of these processes were altered by PFC D_1_ receptor manipulations in a particularly complex manner. Thus, reward sensitivity was not affected by reductions in D_1_ tone but was increased by the lower dose of the D_1_ agonist (Figures 3F, 5C, left). Conversely, D_1_ receptor blockade increased negative feedback sensitivity relative to control conditions, indicating that the decrease in risky choice induced by these treatments was primarily attributable to an increased sensitivity to reward omissions. This effect is similar to that observed after blockade of D_1_ receptors in the nucleus accumbens (Stopper et al., 2013). On the flip side of the curve, D_1_ stimulation had an opposite effect to D_1_ antagonism, reducing lose-shift tendencies (Figures 3G, 5C, right). With respect to D_2_ receptors, blockade or stimulation increased or decreased reward sensitivity, respectively (Figures 3H, 5C, left), whereas, either of these manipulations caused non-significant reductions in negative feedback sensitivity (Figures 3I, 5C, right). Taken together, these data show how distinct aspects of risk/reward decision making can be affected by decreases or increases in mesocortical DA activity in manners that vary considerably across DA receptors. More generally, they further highlight that the specific functions describing how variations in PFC DA activity affect behavior are not uniform across cognitive domains.

**Figure 4:**
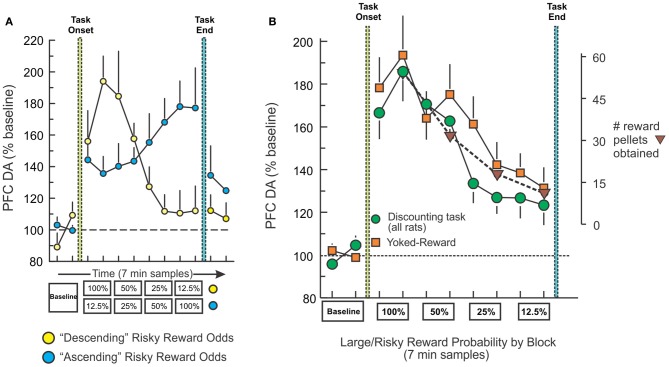
**Fluctuations in PFC DA efflux during decision making track changes in reward rates over time. (A)** Percent change in basal PFC DA extracellular levels obtained from rats trained on the descending (yellow circles) and ascending (blue circles) variants of the probabilistic discounting task, plotted as a function of 7-min sample number. Rats tested on the descending version displayed an initial increase in DA that diminished as large/risky reward probabilities decreased, whereas those trained on the ascending version showed the opposite profile. **(B)** Change in PFC DA efflux for all rats trained on the both variants of the probabilistic discounting task (circles), plotted as a function of probability block. Combined data from rats in the yoked-reward experiment (squares) are also plotted. Triangles represent the number of reward pellets obtained by rats across task blocks. Changes in PFC DA efflux closely tracked changes in the relative amount of food obtain over the course of the session, irrespective of whether rats had to make decisions (task) or it the same amount of reward was delivered passively (yoked). Adapted from St. Onge et al. (2012).

One question that arose from the above-mentioned findings was how do fluctuations in mesocortical DA release relate to modifications in decision basis? To address this, we measured changes in PFC DA efflux with microdialysis in well-trained rats performing the same probabilistic discounting task (St. Onge et al., 2012). PFC DA levels corresponded to changes large/risky reward probabilities irrespective of whether the odds of obtaining the larger reward decreased or increased over a session (Figure 4A, yellow). Thus, when the odds were initially 100% and then decreased across blocks, there was a robust initial increase in PFC DA efflux (~80–90% above baseline) that steadily declined over the session, whereas the opposite profile was observed when the odds were initially poor (12.5%) and subsequently increased (Figure 4A, blue). In this experiment, we included a key, yoked-reward control group consisting of rats that were not required to press any levers or make any decisions, but instead were accustomed to receiving food delivered passively on a schedule similar to rats performing the decision making task. Yoked rats displayed a profile of PFC DA efflux that was nearly identical to that observed during decision making, confirming that the fluctuations in PFC DA transmission during either condition corresponded primarily to changes in the relative rate of reward received (Figure 4B). These findings suggest that dopaminergic afferents to the frontal lobes convey information about changes in the relative amount of reward availability over time, irrespective of whether an organism actually has to do anything to retrieve that reward. However, these data suggest that in situations that require monitoring of changes in rates of reward delivery, dynamic fluctuations in tonic mesocortical DA levels may serve as a reward “running-rate meter,” informing the PFC about changes in reward rates that can aid in adjusting choice accordingly (Niv et al., 2007).

The finding that PFC DA transmission is finely tuned to variations in reward availability provides additional insight into how pharmacological manipulations of DA activity might alter decision making. Thus, interfering with these dynamic signals via D_1_ receptor blockade or stimulation would be expected to cause a discrepancy between the perceived vs. actual rates of reward obtained, leading to corresponding increases and decreases in risky choices. The fact that D_2_ blockade altered decision making in a manner opposite to D_1_ antagonism would suggest that D_2_ modulation of these functions may be less dependent on variations in extracellular PFC DA levels. However, the finding that D_2_ receptor stimulation impaired probabilistic discounting implies that flooding D_2_ receptors may disrupt the ability of a subgroup of PFC neurons to detect changes in PFC DA transmission over time, which may lead to more static patterns of choice.

## PFC DA and cognition: a family of functions

**Figure 5:**
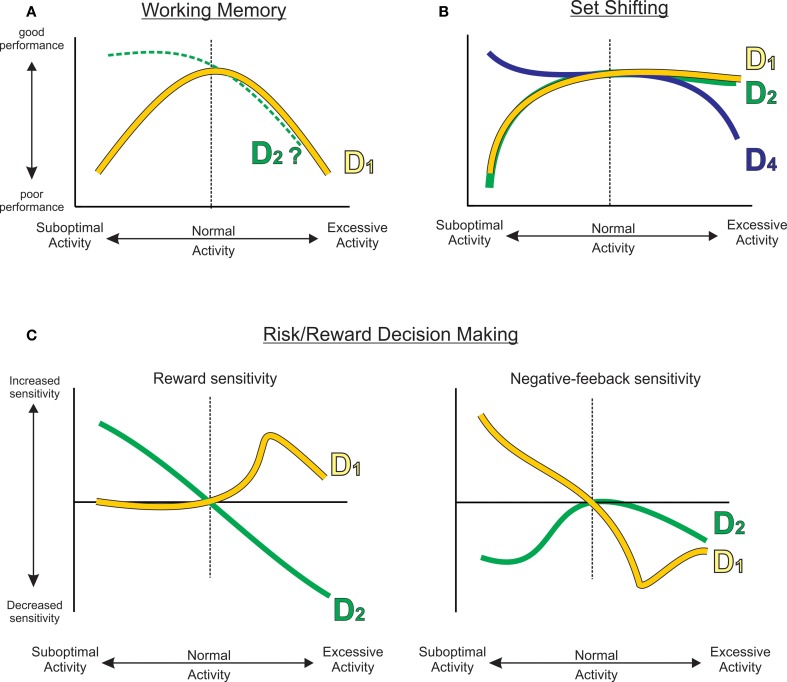
**A family of functions describing how reduced or excessive PFC DA activity can affect different cognitive functions, including (A) working memory, (B) set shifting, or (C) different processes related to risk/reward decision making.** Curves have been extrapolated from empirical data (gray dashed curves) presented in Figures 1–3. The D_2_ working memory curve was extrapolated from Druzin et al. (2000).

The findings reviewed here make it apparent that dopaminergic input to the frontal lobes is an essential component of the neural circuitry mediating a variety of cognitive and executive functions, including working memory, behavioral flexibility, and neuroeconomic processes related to cost/benefit decision making. Each of these requires distinct types of cognitive operations and functional neural circuits. Therefore, it is not surprising that the mechanisms by which DA exerts its effects are not unitary across these functions, but rather, each process relies on different patterns of activation of DA receptors. Thus, PFC D_1_ receptor activity is of primary importance in mediating working memory, whereas, D_1_ and D_2_ receptors act either cooperatively or antagonistically to mediate functions related to behavioral flexibility and reward-related decision making. Moreover, although there is clear evidence that D_1_ receptor modulation of working memory takes the form of an “inverted-U” shaped function, this profile is not necessarily shared by other receptors or across other PFC functions. A survey of the data reviewed here clearly demonstrates that, with respect to PFC DA, the “inverted-U” is not a one-size-fits-all function. Rather, it appears that mesocortical DA exerts its effects via a family of functions, wherein reduced vs. excessive DA activity may produce effects that are monotonic, sigmoidal, biphasic, exponential or polynomial across different cognitive domains (summarized in Figure 5).

The question remains: what are the potential mechanisms underlying these differential effects across cognitive domains? An answer may stem from contemporary theory on how these receptors differentially affect PFC neural network activity (Durstewitz et al., 2000; Seamans and Yang, 2004). D_1_ receptors have been proposed to reduce the influence of weak inputs, stabilizing network activity so that a subset of representation dominates PFC output. Conversely, D_2_ activity attenuates inhibitory influences, allowing PFC neural ensembles to process multiple stimuli and/or representations, placing these networks in a more labile state that may permit changes in representations. With this conceptual framework in mind, it is likely that the cognitive operations underlying different functions would be mediated by distinct patterns of activity within PFC neural networks. Processes related to working memory require stable and persistent patterns of activity encoding information to be used across contexts or time (Goldman-Rakic, 1995; Lapish et al., 2008). The biophysical actions of D_1_ receptors would be best suited for facilitating these patterns of activity. In comparison, shifting between different strategies has been linked to rapid reorganization of PFC neural ensemble activity that encodes different rules and action/outcome contingencies (Durstewitz et al., 2010). It is plausible that upon detection of rule changes, D_2_ receptor activation destabilizes PFC network states, permitting the system to ascertain what the new course of action should be, and once a novel effective strategy has been recognized, stabilization of this new representation would be facilitated by D_1_ receptor activity. Along similar lines, risk/reward decision making requires coordination between various cognitive processes, including those that facilitate flexible responding and action/outcome monitoring over time, which may be mediated by distinct populations of PFC neurons. By striking a fine balance between D_1_ and D_2_ receptor activity, mesocortical DA may help refine cost/benefit decisions between options of varying magnitude and uncertainty, with D_1_ receptors promoting exploitation of current favorable circumstances and D_2_ receptors facilitating exploration of more profitable ones when conditions change. Given these considerations, it is clear that a more comprehensive picture of how DA regulates frontal lobe functioning may be obtained not by painting every cognitive function with the same DA brush, but instead, taking into account the complex myriad of the neurophysiological actions of DA in combination with the neural network activity patterns underlying cognitive operations that subserve different PFC functions. Moreover, the advent of new technologies permitting manipulations of DA transmission in a more temporally and spatially specific manner will undoubtedly yield additional insight into how mesocortical DA regulates different forms of executive functioning. The picture that emerges from future studies of this kind will likely serve to both clarify and at the same time, further complicate our understanding of the functional contribution of PFC DA to cognition.

### Conflict of interest statement

The author declares that the research was conducted in the absence of any commercial or financial relationships that could be construed as a potential conflict of interest.
